# Room temperature phosphorescence from natural wood activated by external chloride anion treatment

**DOI:** 10.1038/s41467-023-37762-9

**Published:** 2023-05-05

**Authors:** Yingxiang Zhai, Shujun Li, Jian Li, Shouxin Liu, Tony D. James, Jonathan L. Sessler, Zhijun Chen

**Affiliations:** 1 Key Laboratory of Bio-based Material Science & Technology, Northeast Forestry University, Ministry of Education, Harbin, 150040 China; 2 Department of Chemistry, University of Bath, Bath, BA2 7AY UK; 3 School of Chemistry and Chemical Engineering, Henan Normal University, Xinxiang, 453007 China; 4 Department of Chemistry, University of Texas at Austin, 105 E 24th Street, A5300 Austin, TX USA

**Keywords:** Optical materials, Environmental impact, Sustainability

## Abstract

Producing afterglow room temperature phosphorescence (RTP) from natural sources is an attractive approach to sustainable RTP materials. However, converting natural resources to RTP materials often requires toxic reagents or complex processing. Here we report that natural wood may be converted into a viable RTP material by treating with magnesium chloride. Specifically, immersing natural wood into an aqueous MgCl_2_ solution at room temperature produces so-called C-wood containing chloride anions that act to promote spin orbit coupling (SOC) and increase the RTP lifetime. Produced in this manner, C-wood exhibits an intense RTP emission with a lifetime of ~ 297 ms (vs. the ca. 17.5 ms seen for natural wood). As a demonstration of potential utility, an afterglow wood sculpture is prepared in situ by simply spraying the original sculpture with a MgCl_2_ solution. C-wood was also mixed with polypropylene (PP) to generate printable afterglow fibers suitable for the fabrication of luminescent plastics via 3D printing. We anticipate that the present study will facilitate the development of sustainable RTP materials.

## Abstract (web summary)

Converting natural wood to room temperature phosphorescent (RTP) materials is a complex process often requiring toxic reagents. Here the authors convert natural wood to a RTP material using external chloride ions from a MgCl2 solution, obtaining a promising luminescent additive material for 3D printing.

## Introduction

Afterglow room temperature phosphorescence (RTP) emission is defined as emission lasting for more than 100 ms after removal of the excitation source^1^. Materials with afterglow RTP emission exhibit long lifetimes, large Stokes shifts, and good signal-to-noise ratios. Often, they can be visualized readily by the naked eye. These attributes have made RTP materials attractive for use in a wide variety of applications, including visual decorations, optical sensing, biological imaging, and information encryption^2–6^. Organic afterglow RTP materials derived from natural sources are of particular interest since they are expected to be sustainable, flexible, biocompatible, and available at scale^7,8^.

To obtain sustainable afterglow RTP materials, two crucial barriers must be overcome^9–11^. First, the triplet excitons of the chromophores inherent in the source material must be effectively populated by facilitating ISC from singlet excitons to triplet excitons, which typically requires efficient spin-orbit coupling (SOC)^12,13^. Second, nonradiative deactivation of the resulting triplet excitons must be suppressed^14–18^.

Guided by these principles, two general strategies for fabricating effective sustainable afterglow RTP materials have been pursued. The first approach relies on converting biomass materials (such as, gelatin, cellulose and rice husks) to carbon dots endowed with efficient SOC; these dots are then confined in an organic matrix to stabilize the triplet excitons^19–21^. Another strategy involves using directly untreated natural materials, such as lignin, gelatin and cellulose, as chromophores incorporated within a rigid matrix^22–25^. However, the fabrication of these sustainable RTP systems generally involves the use of toxic reagents, energy-consuming processes, or complex procedures that are difficult to carry out on large scale. For example, from our previous research we converted wood to structural RTP materials using lignin oxidation assisted by NaOH and concentrated H_2_O_2_^25^. However, drying the concentrated H_2_O_2_-incoporated wood in the oven at high temperatures is very dangerous (potentially explosive). Also, oxidation of lignin destroys the physicochemical stability of the wood^26–29^. As a result, there remains a need for methods that allow afterglow RTP materials to be prepared conveniently and cost-effectively from sustainable sources.

**Fig. 1:**
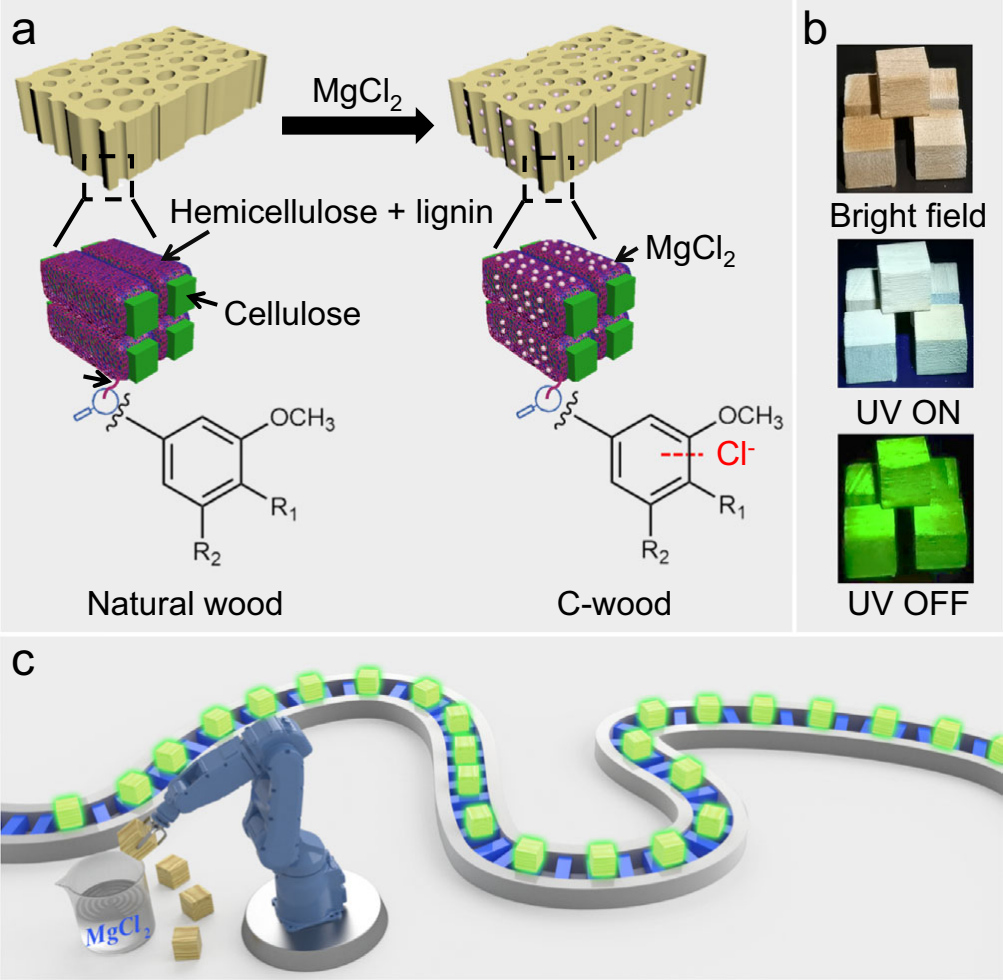
Preparation of C-wood. **a** Schematic showing the preparation of room temperature phosphorescent C-wood from natural basswood. **b** Photographs of C-wood under bright field (upper), UV irradiation (middle), and after switching off the UV light (bottom). Afterglow images were acquired at 120 ms after switching off the UV light. **c** Cartoon illustration of an automatic production line for converting natural wood into an afterglow material.

To address the above challenge, we were drawn to wood. Wood is a largely renewable resource that exhibits short phosphorescence (~17–30 ms), a result attributed to the confinement of lignin within the associated cellulose and hemicellulose matrix^25,30,31^. Previous reports have indicated that the lifetime of phosphorescent chromophores can be enhanced by treating with heavy atom salts^32–34^, certain small molecules^35–39^ or polymers^40–42^. However, these approaches have not been extensively exploited for the creation of natural wood-based afterglow RTP materials in a mild manner. As detailed below, we have now successfully enhanced the lifetime of natural wood from ~17.5 ms to ~297 ms by treating with 1 M aqueous magnesium chloride for 2 sec at room temperature (Fig. 1a). The as-obtained chloride ion-containing wood (C-wood) exhibited ultra-long afterglow emission. With a view toward preparing C-wood rapidly and reproducibly (Fig. 1b), we developed an automatic manufacturing line wherein the source wood was manipulated by robots and subject to immersion in an aqueous MgCl_2_ solution before being placed on the line for transportation and drying (Fig. 1c and Supplementary Movie 1). We also found that C-wood could be converted to afterglow fibers using polypropylene (PP), thus allowing its use in 3D printing.

Generally, natural wood was converted to RTP materials (C-wood) with a lifetime of ~297 ms assisted by external chloride ions. As-prepared C-wood showed great potential as luminescent materials for 3D printing.

## Results

### Afterglow RTP emission of C-wood

**Fig. 2:**
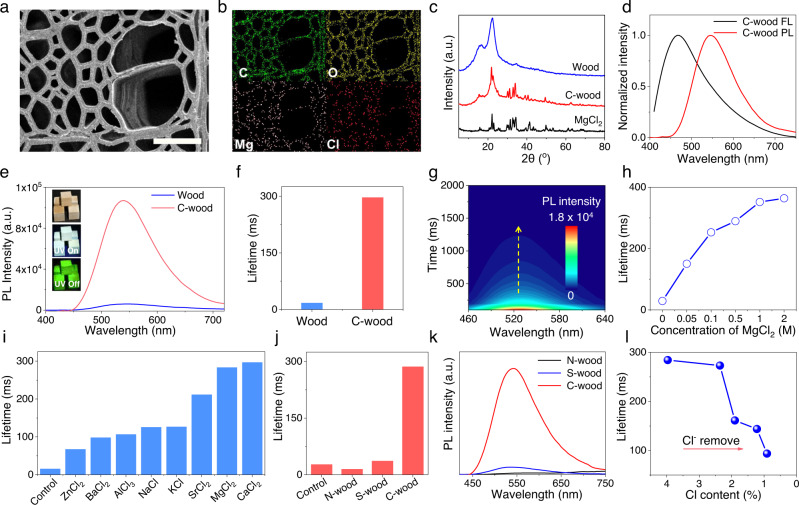
RTP emission of C-wood. **a** Scanning electron microscopy (SEM) images of C-wood; scale bar = 50 μm. **b** Elemental mapping of C-wood: C, O, Mg and Cl. **c** X-ray diffraction (XRD) pattern of wood (blue line), C-wood (red line) and MgCl_2_ (black line). **d** Fluorescence (FL, black line) and Phosphorescence (PL, red line) spectra of C-wood treated with 1 M aqueous MgCl_2_ at room temperature. **e**, Phosphorescence spectra of C-wood (red line) and natural wood (blue line) at room temperature. Afterglow images were acquired at 120 ms after switching off the UV light. **f** Lifetime of wood and C-wood. **g** Time-dependent phosphorescence emission of C-wood. **h**, RTP lifetime of C-wood treated with different concentration of MgCl_2_. **i** Lifetime of C-wood prepared using different salts; the concentration of chloride anions was 2 M in all cases. **j** Lifetime of C-wood (Basswood treated with 1 M MgCl_2_), S-wood (Basswood treated with 1 M MgSO_4_) and N-wood (Basswood treated with 1 M Mg(NO_3_)_2_) at room temperature. **k** RTP spectra of C-wood (red line), S-wood (blue line) and N-wood (black line). **l** Effect of Cl content on the C-wood RTP lifetime. Excitation wavelength = 365 nm.

In this study, basswood was chosen as a representative natural wood. It was converted to C-wood by soaking in 1 mol L^−1^ (M) aqueous MgCl_2_ for 2 s at room temperature. The MgCl_2_ loading of C-wood is 8.5%, determined by changes in mass. An automatic manufacturing line was also developed for preparing the wood in a convenient and rapid manner (Supplementary Movie 1). SEM imaging studies confirmed that the resulting C-wood maintained a porous structure analogous to that of the original source wood (Fig. 2a and Supplementary Fig. 1). Elemental mapping of the C-wood confirmed that C, O, Mg and Cl are evenly distributed within the wood cell walls (Fig. 2b). Moreover, the XRD pattern for the C-wood produced in this way exhibited the characteristic signals of natural wood and MgCl_2_, leading us to conclude that treatment with MgCl_2_ did not alter the basic structure of the source material (Fig. 2c). Specifically, the natural wood exhibited a strong signal in 20^o^, which was attributed to Type I cellulose in the wood matrix^43^. Treatment using MgCl_2_ did not change the crystal structure of the natural wood. Spectroscopic analyses of the C-wood revealed a fluorescence emission feature centered at 465 nm and strong afterglow RTP emission centered at 545 nm with a lifetime of ~ 297 ms and the quantum yield was ~ 4.7% (Fig. 2d to f, Supplementary Fig. 2 and Supplementary Movie 2). To confirm reproducibility, we prepared 5 samples under the same conditions and measured their lifetimes with 5 repeat measurements being made for each sample (Supplementary Fig. 3). Concordant and reproducible results were obtained. In contrast, untreated wood exhibited a weak afterglow emission and a relatively short lifetime (~ 17.5 ms) (Supplementary Fig. 2). Time-resolved spectroscopic^44–46^ analyses confirmed that C-wood produces a long-lasting and stable afterglow phosphorescence feature centered at 545 nm following photoexcitation at 365 nm, with this emission remaining detectable for as long as 1100 ms (Fig. 2g). The lifetime of the C-wood could be tuned by varying the concentration of the MgCl_2_ solution with which the native wood was treated. For instance, the lifetime of C-wood could be increased to ca. 364 ms when 2 M MgCl_2_ was used (Fig. 2h). Native wood treated in an analogous way with different monovalent, divalent or trivalent chloride anion salts at equivalent chloride anion concentrations (2 M), including AlCl_3_, ZnCl_2_, KCl, NaCl, BaCl_2_, SrCl_2_ and CaCl_2_, also exhibited enhanced lifetimes when compared with the untreated wood (control group) (Fig. 2i). To exclude the effect of varied pH of these solution on the RTP emission of wood, natural wood was treated using the corresponding pH mediated by acid (CH_3_COOH) or base (NH_3_·H_2_O). The treated wood did not show enhanced RTP lifetime (Supplementary Fig. 4 and Supplementary Fig. 5).

Samples were also prepared by treating natural basswood with Mg(NO_3_)_2_ and MgSO_4_. Basswood treated with Mg(NO_3_)_2_ (N-wood) and MgSO_4_ (S-wood) exhibited weak phosphorescence emission and shorter lifetimes of 4.9 ms and 36.6 ms, respectively (Fig. 2j and k). The reduced lifetime of N-wood was proposed to reflect the quenching effect of NO_3_ anions^47,48^. We thus conclude that the chloride anion plays a determinative role in enhancing the RTP lifetime. Other halide anion salts, namely KBr, MgBr_2_, and KI, were also evaluated; again, an enhancement in the RTP lifetime was observed (Supplementary Fig. 6–8). However, for reasons of ease, efficacy, and cost we focused on MgCl_2_ in the present study.

To verify that the MgCl_2_ was crucial for RTP emission, C-wood was washed with water to remove the MgCl_2_. After washing, the lifetime of C-wood decreased from ca. 284 ms to approximately 94 ms with a concomitant decrease in the elemental chlorine content from 3.97% to 0.91%, determined by XPS analysis (Fig. 2l and Supplementary Fig. 9). The effect of environmental humidity on the phosphorescence lifetime of C-wood was also evaluated. The humidity can quench the triplet excitons^49,50^. As a result, the C-wood was easily quenched by humidity. It was found that the phosphorescence lifetime decreased as the humidity increased. For instance, the phosphorescence lifetime dropped to 5.89 ms when the humidity reached 90% (Supplementary Fig. 10). The phosphorescence lifetime recovered after the sample was dried. Only a modest degradation in the RTP lifetime of the dried C-wood was observed over the course of repeated “humidifying-dehumidifying” cycles (Supplementary Fig. 11). However, the problem can be solved by coating the RTP wood with a hydrophobic wax. After coating with wood wax, the lifetime of wood did not decrease upon changes of humidity (Supplementary Fig. 12).

### Mechanism and universal design

To further understand the role of lignin, delignified wood was prepared^51^. As expected, delignified wood exhibited very short phosphorescence lifetime (~2.1 ms). Introducing MgCl_2_ to the delignified wood did not obviously enhance its RTP lifetime (~2.3 ms) (Supplementary Fig. 13). Additionally, embedding technical lignin, such as, alkali lignin (AL) and lignin sulfonate (LS-Na) in cellulose pulp resulted in RTP emissions with lifetimes of 127.5 ms and 141.7 ms, respectively. In addition, the value increased to 554.7 ms and 356.1 ms, respectively upon addition of MgCl_2_ (Supplementary Fig. 14 and Supplementary Fig. 15). All these results confirmed that lignin is the RTP chromophore in the wood matrix and could be activated by external chloride anions.

**Fig. 3:**
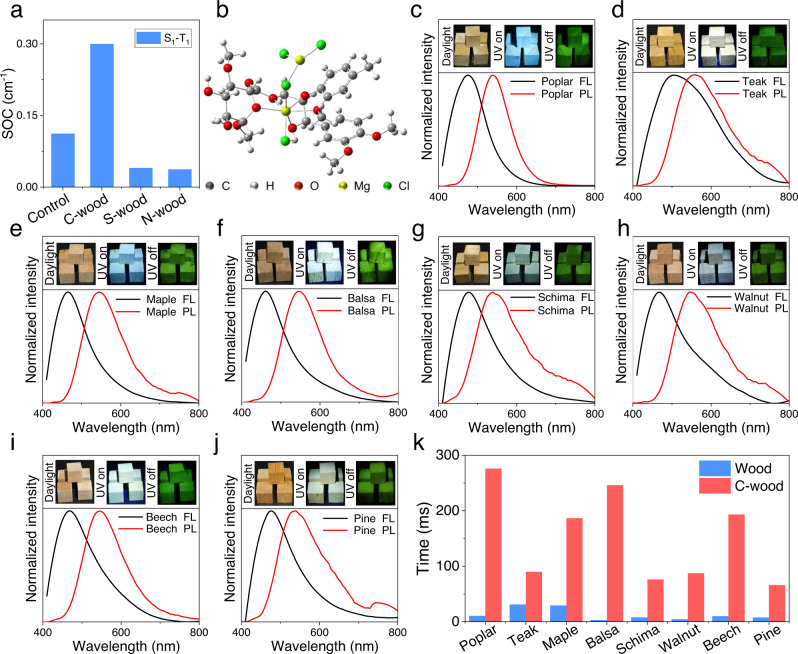
Mechanistic studies and tests of generality. **a** Spin orbit coupling (SOC) values between the S_1_ and T_1_ states calculated for model C-wood, S-wood, N-wood and Control (natural wood) samples. **b** Simulated structure of a C-wood model. **c–j** Fluorescence (black line) and phosphorescence (red line) spectra of poplar, teak, maple, balsa, schima, walnut, beech and pine treated with 1 M aqueous MgCl_2_ at room temperature. The inset shows C-wood before (left) and after (right) turning off the excitation source (a 365 nm UV lamp). All afterglow images in **c–j** were acquired at 120 ms after switching off the UV light. **k**, RTP lifetime of poplar, teak, maple, balsa, schima, walnut, beech, pine and the corresponding C-wood forms. Excitation wavelength = 365 nm.

To gain insights into the determinants underlying the observed increase in the RTP lifetime seen for C-wood, theoretical calculations were performed. Here, a model unit (Supplementary Fig. 16) was employed for lignin-carbohydrate complexes in the wood^52^. As determined by the high-resolution XPS spectral studies, there are two types of Cl^–^ in C-wood, namely Cl^–^ from free MgCl_2_ and Cl^–^ from coordinated MgCl_2_^53^ (Supplementary Fig. 17). Both types of Cl^–^ anions were considered in the calculations (Fig. 3b). The distance between the Cl^–^ (from coordinated MgCl_2_) and the closest π surface was found to vary between 4.6 and 5.0 Å, leading us to conclude that the chloride anion−π interactions are at best modest (Supplementary Fig. 18). However, the corresponding Cl^–^···π distance (for the free MgCl_2_) proved to be 3.56 Å. This relatively close contact was expected to facilitate SOC promoted by the chloride anion. (Fig. 3b and Supplementary Fig. 18). The theoretical calculations also provided support for the notion that the SOC constant for C-wood (0.30 cm^−1^) was higher than the value of natural wood (0.11 cm^−1^), S-wood (0.04 cm^−1^) and N-wood (0.037 cm^−1^) (Fig. 3a). A strong SOC facilitates ISC and thus serves to enhance the RTP emission. Spin-orbit coupling values are positively correlated with generation of singlet oxygen^54–56^. Therefore, we determined the generation of singlet oxygen by C-wood and natural wood using 9,10-anthracenediyl-bis(methylene)-dimalonic acid (ABDA) as a singlet oxygen probe^57^. The results indicated that C-wood exhibited a faster and more significant generation of singlet oxygen than natural wood, suggesting that C-wood has an enhanced SOC value (Supplementary Fig. 19). On the bases of these analyses, we propose that it is the Cl^–^···π interactions in the MgCl_2_-treated samples that give rise to the observed long afterglow emission.

To test whether the present strategy could be expanded to include other types of wood, samples of poplar, teak, maple, balsa, schima, walnut, beech and pine wood were treated with MgCl_2_ using the same protocol as described above. In all cases, the resulting samples were found to fluorescence blue when irradiated at 365 nm and produce a long-lived yellow-green phosphorescence.

**Fig. 4:**
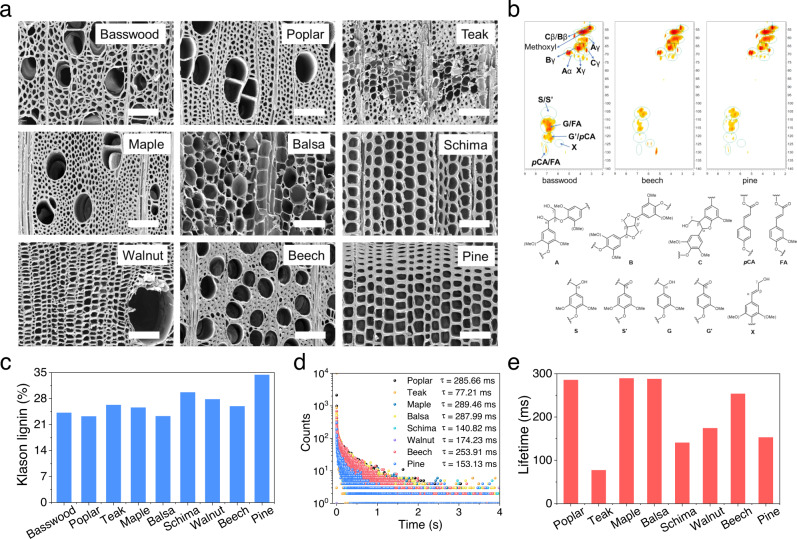
Mechanism for different RTP behavior from various wood species. **a** SEM image of different natural wood, scale bar = 100 μm. **b** The 2D HSQC NMR spectra of lignin from basswood, beech and pine (DMSO-d_6_, 200 mg mL^−1^). **c** Klason lignin content of basswood, poplar, teak, maple, balsa, schima, walnut, beech and pine. RTP decay profiles (**d**) and RTP lifetime (**e**) of poplar, teak, maple, balsa, schima, walnut, beech and pine treated with 1 M CaCl_2_. Excitation wavelength = 365 nm.

Interestingly, the C-wood made from different types of wood exhibited different emission spectra and lifetimes. To better understand why this was the case, the loading and inherent physiochemical properties of natural wood were analyzed. Mass analysis indicated that the wood had different MgCl_2_ loading amount although they were treated with the same concentration of MgCl_2_ solution (Supplementary Fig. 20). This was attributed to different adsorbance capacities of the wood caused by the various porous structures^58–61^ (Fig. 4a). Notably, C-wood made from basswood and schima wood displayed different spectra and lifetime although they had similar MgCl_2_ loading. This was associated with the inherent physiochemical properties of lignin in the wood, which is crucial for the RTP emission. Generally, lignin consists of three aromatic units, guaiacyl lignin (G), syringyl lignin (S) and para-hydroxy-phenyl lignin (H) linked by C-O-C and C-C bond. The ratios of these aromatic units and linkages varies between wood species^62,63^. These different molecular structures, supramolecular aggregates and concentrations of lignin can result in different photoluminescence properties^64,65^. To clarify the difference of lignin in these woods, the content of lignin was characterized using the Klason method^66^. As expected, the content of lignin in these woods was different and varied from 23.2% to 34.4% (Fig. 4c). Also, taking basswood, beech and pine as examples, 2D HSQC NMR analysis was performed^67^. The results indicated that lignin in these woods had different G/S/H ratios and structures (Fig. 4b). All these results suggest that a different loading of MgCl_2_ and the inherent physiochemical properties of lignin contribute to the various emission spectra and lifetimes.

The corresponding afterglow RTP lifetimes for the treated poplar, teak, maple, balsa, schima, walnut, beech and pine wood samples were 275.87 ms, 89.94 ms, 186.41 ms, 246.10 ms, 76.08 ms, 87.34 ms, 193.04 ms and 66.04 ms, respectively. These RTP effects could be observed easily by the naked eye after the UV lamp was switched off (Fig. 3c-k). We thus suggest that MgCl_2_ treatment provides a general means of preparing afterglow materials from wood. Additionally, the long-lived RTP lifetime of C-wood using different wood species was also investigated (Fig. 4d, e). The corresponding afterglow RTP lifetimes for the treated poplar, teak, maple, balsa, schima, walnut, beech and pine wood samples were 285.66 ms, 77.21 ms, 289.46 ms, 287.99 ms, 140.82 ms, 174.23 ms, 253.91 ms and 153.13 ms, respectively.

### Potential applications

**Fig. 5:**
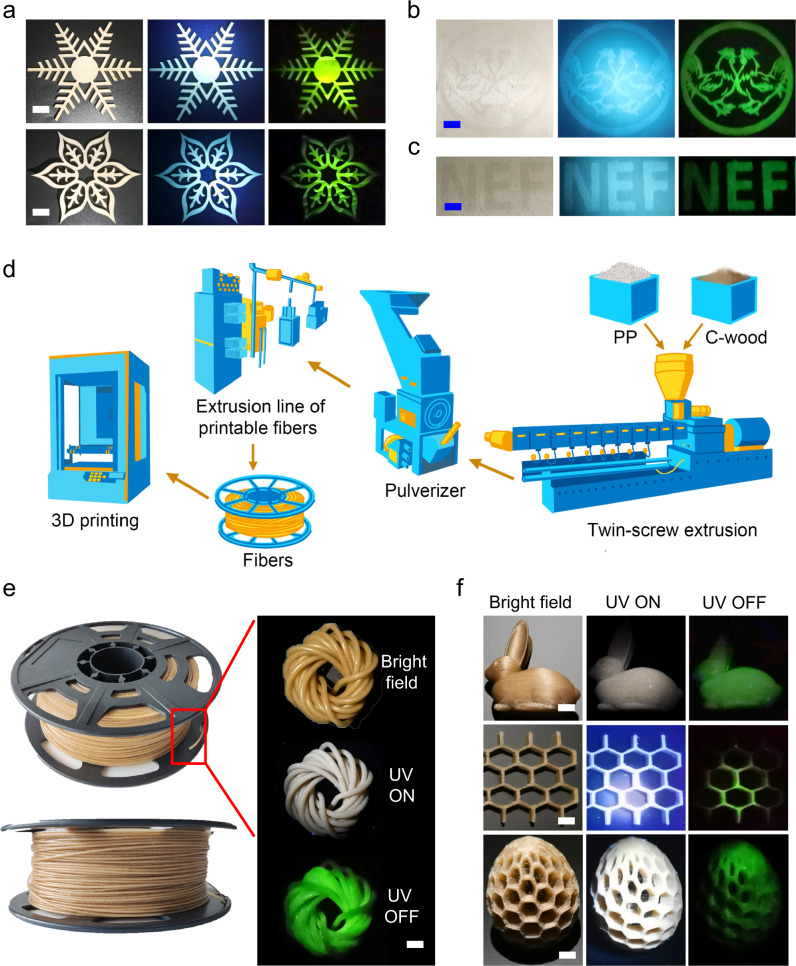
RTP emission of C-wood products. **a**, Photographs of C-wood sculpture before (middle) and after (right) turning off a 365 nm UV lamp; scale bar = 1 cm. **b** and **c** Photographs of C-wood pattern before (middle) and after (right) turning off a 365 nm UV lamp; scale bar = 1 cm. Afterglow images in a-c were acquired at 120 ms after switching off the UV light. **d**, Schematic illustration of fabrication of printable afterglow plastics from C-wood and polypropylene (PP). **e** Photographs of printable fiber before (middle) and after (bottom) turning off a 365 nm UV lamp; scale bar = 1 cm. **f** Photographs of printed rabbit, pinecones, and honeycomb before (middle) and after (right) turning off a 365 nm UV lamp; scale bar = 1 cm. Afterglow images in e-f were acquired at 140 ms after switching off the UV light.

Sustainable indoor lighting materials represent an obvious and substantial requirement. The present C-wood approach could play a role in this regard. In order to evaluate this possibility, a basswood sculpture was sprayed with a MgCl_2_ solution. In accord with our expectations, the treated sculpture exhibited afterglow emission (Fig. 5a). Moreover, patterned structures could be obtained by spraying MgCl_2_ through a template onto the surface of a basswood surface (Fig. 5b, c).

Luminescent plastics are another material of interest. They exhibit promise for use in energy-storage applications^68^, LED production^69^ and information storage^50^. 3D printable luminescent plastics have received particularly attention in this context due to the ever-increasing demand for irregularly shaped materials. To test whether the present C-wood could be used to prepare afterglow luminescent plastics useful for 3D printing, we mixed C-wood with polypropylene (PP). Specifically, C-wood powder (10 g) was mixed with PP (990 g) to provide C-wood/PP bulk composites (cf. Supporting Information for experimental details). Subsequently the C-wood/PP bulk composite was processed to generate printable fibers using a screw extruder (Fig. 5d, e). The fibers exhibited a tensile strengthen of 25.63 Mpa (Supplementary Fig. 21). These fibers were then printed into representative shapes, such as a rabbit, honeycomb, and pinecone, (Fig. 5f). All the resulting constructs exhibited long afterglow emission following photoexcitation. Moreover, the printed sample did not show any lifetime decrease after storage for 3 months (Supplementary Fig. 22).

## Discussion

**Fig. 6:**
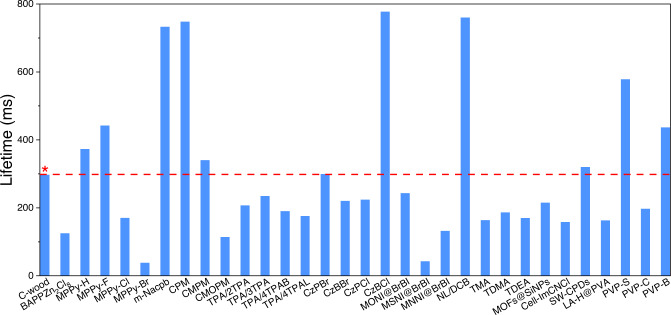
Comparison of RTP lifetime between C-wood (∗) and the reported materials. (BAPPZn_2_Cl_8_;^46^ MPPy-H, MPPy-F, MPPy-Cl and MPPy-Br;^75^ m-Nacpb;^76^ CPM, CMPM and CMOPM;^77^ TPA/2TPA, TPA/3TPA, TPA/4TPAB and TPA/4TPAL;^78^ CzPBr, CzBBr, CzPCl and CzBCl;^79^ MONI@BrBI, MSNI@BrBI and MNNI@BrBI;^80^ NL/DCB;^81^ TMA, TDMA and TDEA;^82^ MOFs@SiNPs;^83^ Cell-ImCNCl;^84^ SW-CPDs;^85^ LA-H@PVA;^86^ PVP-S, PVP-C and PVP-B^87^).

In summary, we have demonstrated that natural woods may be converted into afterglow RTP materials by simply treating with aqueous MgCl_2_. The lifetime of natural basswood could be increased by ~17-fold (to give an RTP lifetime of ~ 297 ms) by treating with aqueous 1 M MgCl_2_ for 2 sec at room temperature. This increase is ascribed to the enhancement in SOC produced by the embedded chloride anions. In operational terms, it is important to note that no significant energy-consuming steps or toxic reagents were required for the conversion of native wood to C-wood. Further, C-wood in combination with PP could be employed to produce afterglow printable fibers, which could then be 3D printed to create a variety of RTP constructs. We believe our materials exhibit many advantages for practical applications, when compared with traditional RTP materials: a. The raw sources used for the RTP materials were sustainable and renewable wood, which could be obtained in a large scale at a low price, b. The method of fabricating the RTP materials from natural wood was simple and mild and does not generate toxic byproducts during the preparation; c. Additionally, our wood-derived RTP materials could be easily converted to powders, films and structural materials suitable for any practical applications^25,70^. Considering these benefits, we believe that the present strategy could find use in a range of application areas where RTP materials might prove useful, including in anti-counterfeiting operations, flexible display production, and generation of textiles and luminescent coatings. Research into C-wood remains in its infancy and as such shortcomings associated with practical applications exist, particularly when compared with other emerging phosphorescent materials, such as, molecular crystals, guest-host molecular systems, metal-organic frameworks and polymer composites, which are regarded as promising replacements for traditional inorganic bulk phosphorescent materials^11,71–74^. For example, the lifetime of C-wood does not show any significant improvements when compared with currently reported materials. However, C-wood does exhibit comparable lifetime with many of these materials (Fig. 6)^46,75–87^. As such an important future goal will be to enhance the lifetime of C-wood. Additionally, the RTP wavelength of the reported phosphorescent materials can be tuned trough targeted synthesis^88^. However, in the case of C-wood, we are limited by the naturally available structure of lignin, as such the RTP wavelength of C-wood cannot be easily modified. One possibility for future development would be to collaborate with plant physiologist to obtain modified lignin via genetic engineering^89^ and grow wood with targeted RTP emission spectra after Cl^-^ modification. As such in the future it may be possible to prepare C-wood with different RTP wavelengths using modified natural wood.

## Methods

### Preparation of C-wood, N-wood and S-wood

The wood samples were immersed in a 1 M aqueous MgCl_2_ solution (or aqueous solution of other salts that were 2 M in Cl^–^). After immersing for 2 s, the wood sample was removed from the MgCl_2_ solution (or the aqueous solution of other salts) and dried at 105 °C for 60 min to give C-wood. N-wood and S-wood were obtained by replacing the MgCl_2_ with 1 M aqueous Mg(NO_3_)_2_ or 1 M aqueous MgSO_4_ solution, respectively, following the same procedure.

### Preparation of lignin for 2D-HSQC NMR analysis

Choline chloride (ChCl, 2.0 g) and lactic acid (12.9 g) were mixed together and heated at 80 °C until the solid powders completely disappeared to obtain a deep eutectic solvent (DES). After that, the mixture was cooled in a desiccator. Prior to DES treatment, the wood samples were air-dried and milled to a specific size (mesh 40). After that, dry wood powders (1 g) were added to DES (10 g). The reaction mixture was maintained at 145 °C for 6 h. The mixture was then cooled down and the DES soluble fractions were separated by filtration assisted by ethanol washing (30 mL). Water (100 mL) was then added to the filtrate to precipitate the lignin. The as-obtained lignin was collected after centrifugation and freeze-dried for NMR analysis.

### Klason method

Klason lignin is the insoluble residue portion after removing the ash by concentrated acid hydrolysis of the plant tissues, which is also an intuitive method for the determination of lignin content in plants. Specifically, the wood powders (1 g) were firstly washed using a mixture of benzene (67 mL) and ethanol (33 mL). After that, the wood was dried at 105 °C for 60 min. The as-dried wood was treated with concentrated sulfuric acid (72%, 15 mL) at 30 °C for 4 h. Then, water (545 mL) was added to the reaction. The reaction was further refluxed for 2 h. Finally, the reaction mixture was filtered, and as-obtained solid powders were washed by water and dried at 105 °C for 60 min to give lignin.

### Preparation of printable fibers

C-wood powder was prepared by immersing basswood powder in 2 M Cl^-^ for 2 s. As-obtained C-wood powders were then dried at 105 °C for 2 h. Preparation of C-wood/PP blends: 990 g PP and 10 g C-wood powder were physically mixed. After that, the mixture was subjected to extrusion processing and pelleting at 190 °C to give C-wood/PP blends. This C-wood/PP bulk composite material was then melted and pulverized at a speed of 30 rpm. The pulverized C-wood/PP powders were further treated using a single-screw wire extruder at a temperature of 175 °C, which was subsequently wound on a wire reel. The fibers were printed into models, such as, rabbit, honeycomb and pinecones, using standard 3D printing methods.

## Supplementary information

Supplementary Information

Description of Additional Supplementary Files

Supplementary Movie 1

Supplementary Movie 2

## Data Availability

All relevant data are included in this article and its Supplementary Information files. Source data are provided with this paper.

## Source data

Source Data
