# Supplementary material for: Room temperature phosphorescence from natural wood activated by external chloride anion treatment
Source: Nat Commun. 2023 May 5;14:2614. doi: 10.1038/s41467-023-37762-9 (PMC10162966; doi:10.1038/s41467-023-37762-9)
Supplement: Supplementary file 1 — Supplementary Information [file 41467_2023_37762_MOESM1_ESM.pdf]

Supplementary Information

Room temperature phosphorescence from natural wood activated by external chloride anion treatment

Yingxiang Zhai[a], Shujun Li*[,a], Jian Li[a], Shouxin Liu[a], Tony D. James*[,b,c], Jonathan L. Sessler*[,d], Zhijun Chen*[,a]

[a]Key Laboratory of Bio-based Material Science & Technology, Northeast Forestry University, Ministry of Education, Harbin 150040, China. [b]Department of Chemistry, University of Bath, Bath, BA2 7AY, UK. [c]School of Chemistry and Chemical Engineering, Henan Normal University, Xinxiang 453007, China. [d]Department of Chemistry, University of Texas at Austin, 105 E 24th Street, A5300, Austin, USA.

*e-mail: lishujun@nefu.edu.cn; chenzhijun@nefu.edu.cn; T.D.James@bath.ac.uk; sessler@cm.utexas.edu.

Supplementary Methods

Materials

All wood used in this article was purchased from Alibaba (Hangzhou, China). Polypropylene (PP, T30S) resin was purchased from China National Petroleum Corporation (Daqing, China). $MgCl_2$ (>99%), $CaCl_2$ (>99%), $ZnCl_2$ (>98%), $BaCl_2$ (>98%), $AlCl_3$ (>99%), NaCl (>99%), KCl (>99%), SrCl2 (>99%), $Mg(NO_3)_2$ (>99%), $MgSO_4$ (>99%), KBr (>99%), $MgBr_2$ $6H_2O$ (>98%), KI (>99%), Choline chloride (ChCl, >98%) and L-Lactic Acid (>85%) were purchased from Aladdin (Shanghai, China). Alkali lignin and lignin sulfonate (average Mw ~52,000, average Mn ~7,000) were purchased from Sigma-Aldrich (Shanghai, China). CH_3COOH (>99%), $NH_3{\cdot}H_2O$ (25%~28%, w/w), NaOH (>96%), Na_2SO_3 (>97%), H_2O_2 solution (30%, w/w) and H_2SO_4 (95%~98%) were purchased from Kermel Chemical Industry (Tianjin, China). 9,10-anthracenediyl-bis(methylene)-dimalonic acid (ABDA, >90%) was purchased from Macklin Biochemical Co., Ltd (Shanghai, China). Deionized water was produced using a Smart-RO ultrapure water system (Hitech Instruments Co., Ltd., Shanghai, China). Components for the custom-built apparatus were purchased from Taobao (Alibaba, Hangzhou, China).

Characterization

Fluorescence spectra, afterglow spectra, and lifetime decay curves were recorded using a FLS1000 photoluminescence spectrometer (Edinburgh Instruments, Livingston, UK) equipped with a xenon lamp and a one-microsecond lamp (detector: photoelectric multiplier, 200 nm < λ < 1700 nm). Afterglow emission spectra were recorded after a 10 ms delay. Fourier transform infrared (FT-IR) spectra were recorded using a Nicolette 6700

FT-IR spectrometer (ThermoFisher Scientific, USA) from 400 cm^{-1} to 4000 cm^{-1}, using the attenuated total reflection (ATR) method. The pH value was measured by pH meter (FiveEasy Plus, FE28, Shanghai, China). X-ray photoelectron spectroscopy (XPS) was carried out using an ESCALAB 250x X-ray photoelectron spectrometer (American Thermo Co., Ltd., Waltham, Massachusetts, USA) equipped with a monochromatic Al Kα X-ray source. 2D HSQC NMR spectra was recorded on a Bruker 700 MHz NMR spectrometer with TMS as the internal standard. The models were printed by a 3D printer (FT300 DIY Dual nozzle 3D printer_Flythinking Technology Co., Ltd). The tensile strength of printable fibers was measured using a UTM2203 universal testing machine (Shenzhen SUNS Technology Stock Co. Ltd., Shenzhen, China), with a 100 N load cell.

Simulation methods

The geometries of the isolated molecule and the structures of the supramolecular complexes formed upon treatment molecule with $MgCl_2$, $Mg(NO_3)_2$ and $MgSO_4$ were optimized using the semi-empirical method GFN-Xtb[1,2]. For each complex, one hundred possible structures were generated randomly and then searched using a molecular program described in the literature (Tian Lu, Molclus Program, Version 1.9.9.2). These clusters were then optimized in a preliminary manner using a xTB program[3]. After this preliminary structural optimization, the cluster for any given salt with the lowest energy was selected as the most likely structure. This selected structure was then optimized using density functional theory (DFT) using the PBE0 functional[4] and the def2-SVP[5] basis set. The DFT-D3 dispersion correction method was used in all calculations. To probe the photophysical properties of the putative supramolecular complexes, their excited

electronic structures were calculated at the PBE0-D3/def2-TZVP level using time-dependent density functional theory (TDDFT). The spin-orbit coupling (SOC) matrix elements were calculated using the spin-orbit mean-field (SOME) method based on the excited state wave functions obtained from the TDDFT calculations. These calculations were performed using the ORCA 4.2.1 program[6,7]. The frontier molecular orbitals were rendered using the Visual Molecular Dynamic program (VMD)[8].

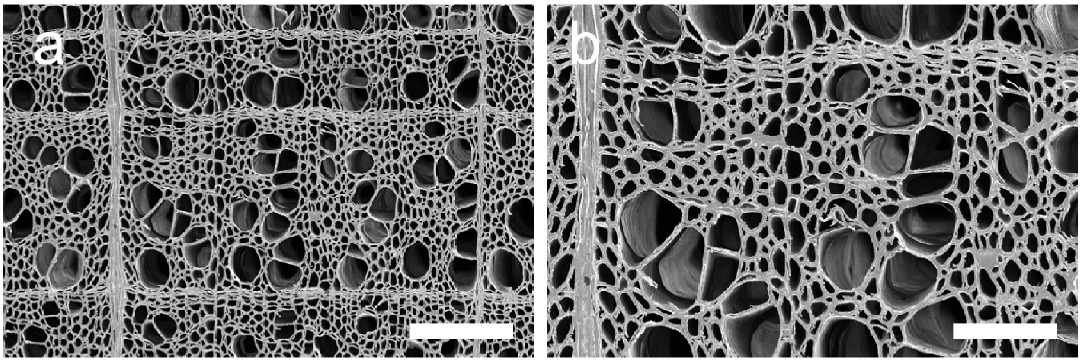

Supplementary Fig. 1. SEM image of natural basswood (a: scale bar = 200 μm, b: scale bar = 100 μm).

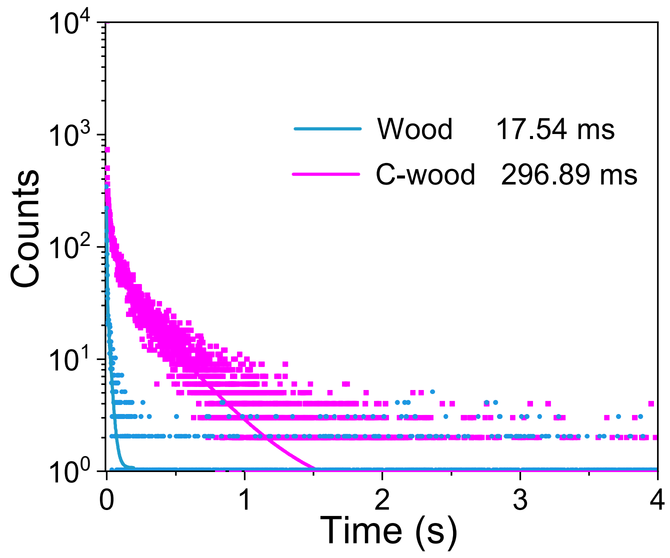

Supplementary Fig. 2. RTP decay profiles of native basswood and C-wood; excitation wavelength = 365 nm.

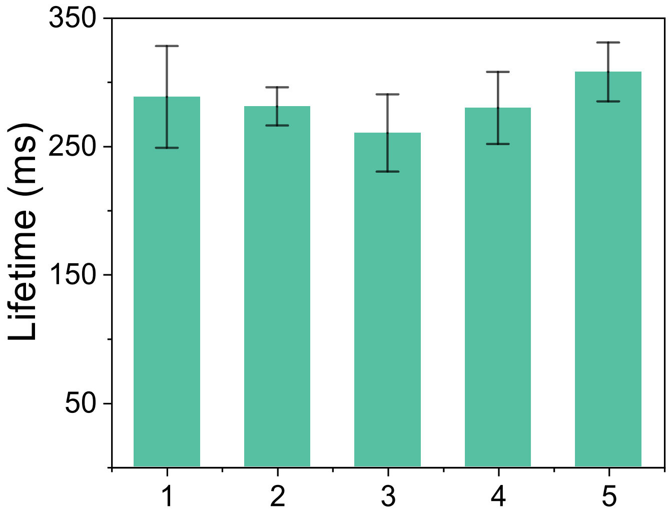

Supplementary Fig. 3. Luminescent lifetimes of C-wood (Error bars indicated standard deviations of five separate measurements).

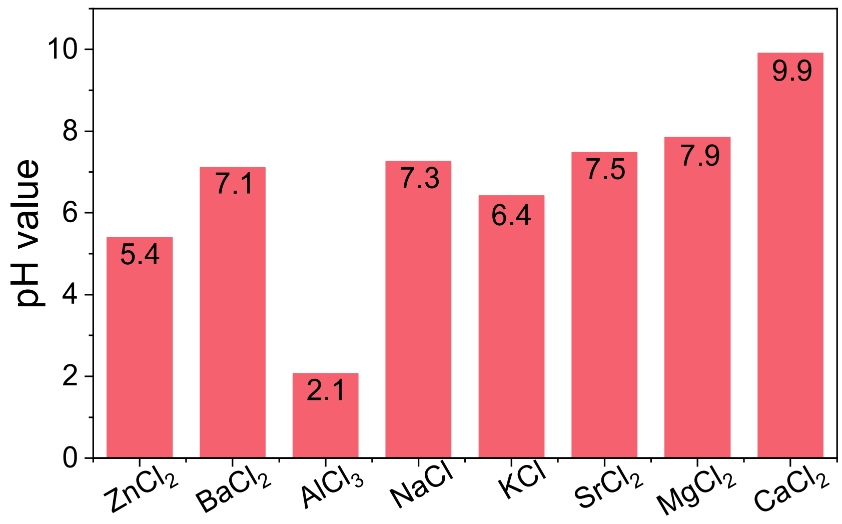

Supplementary Fig. 4. The corresponding pH value of different types of salt in water (2M Cl^-).

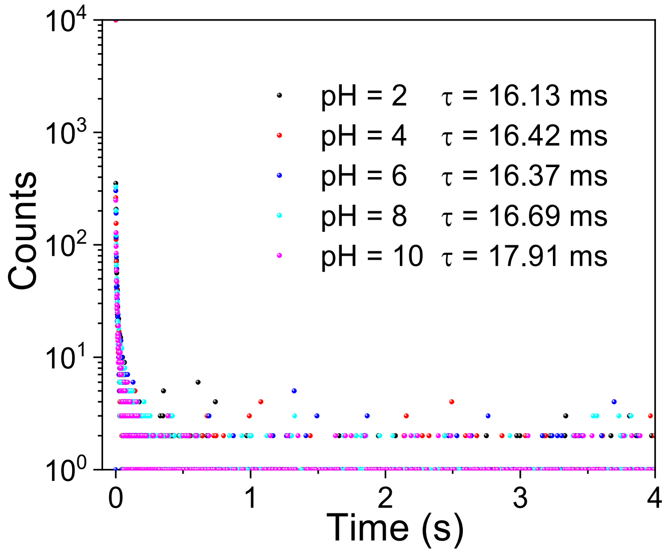

Supplementary Fig. 5. RTP decay profiles of nature wood treated at different pH.

(Sample for measurement: Wood was firstly immersed in the solution for 2s and then dried at 105 °C, CH_3COOH (>99%) and $NH_3 \cdot H_2O$ (25%~28%, w/w) were used for pH

adjusting)

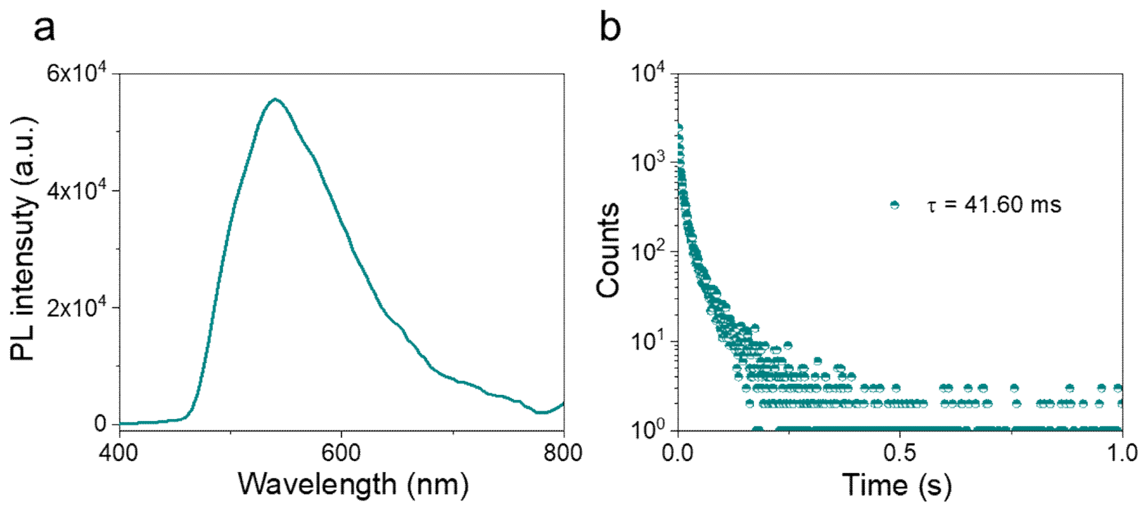

Supplementary Fig. 6. Afterglow emission spectra (a) and lifetime (b) of C-wood prepared using KBr. Excitation wavelength = 365 nm.

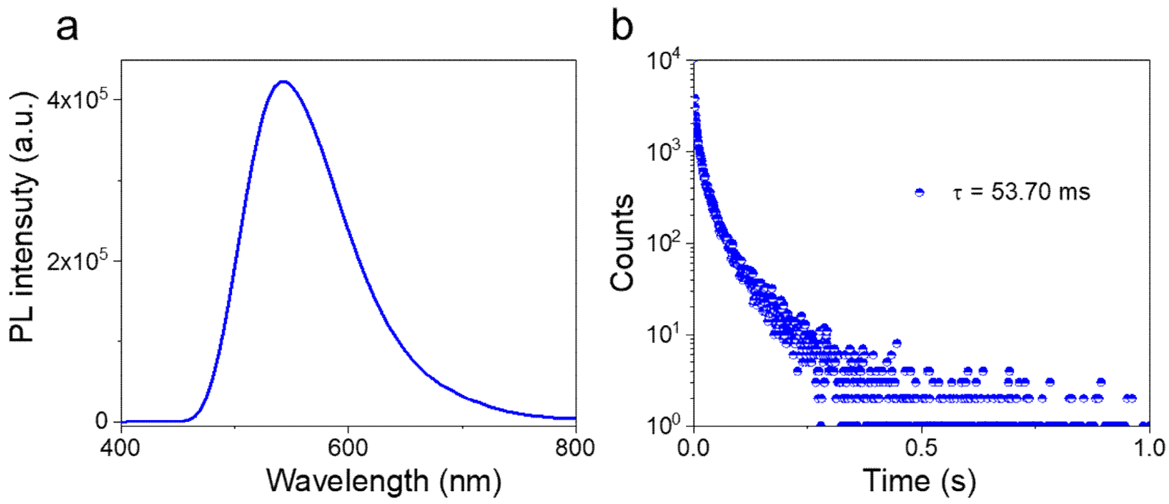

Supplementary Fig. 7. Afterglow emission spectra (a) and lifetime (b) of C-wood prepared using $MgBr_2$. Excitation wavelength = 365 nm.

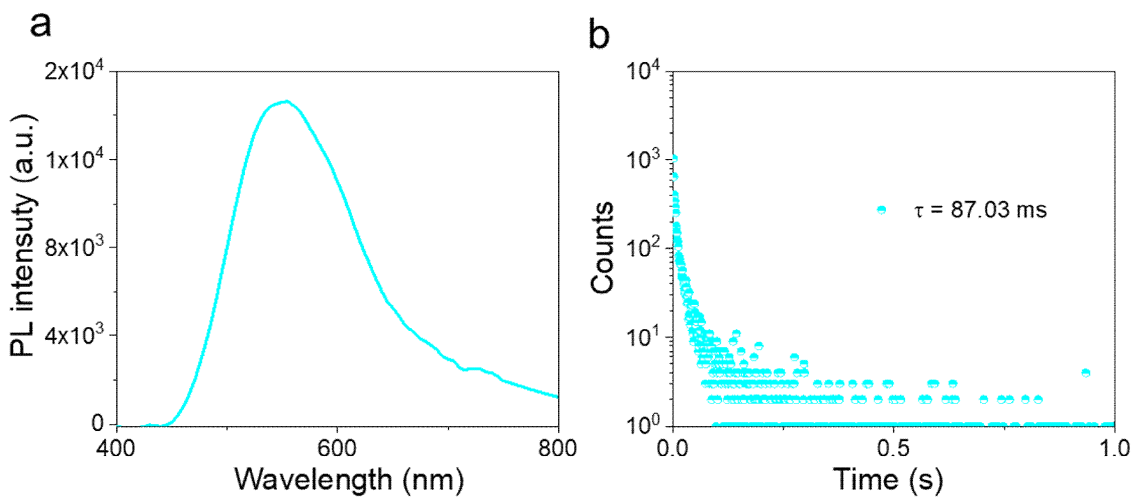

Supplementary Fig. 8. Afterglow emission spectra (a) and lifetime (b) of C-wood prepared using KI. Excitation wavelength = 365 nm.

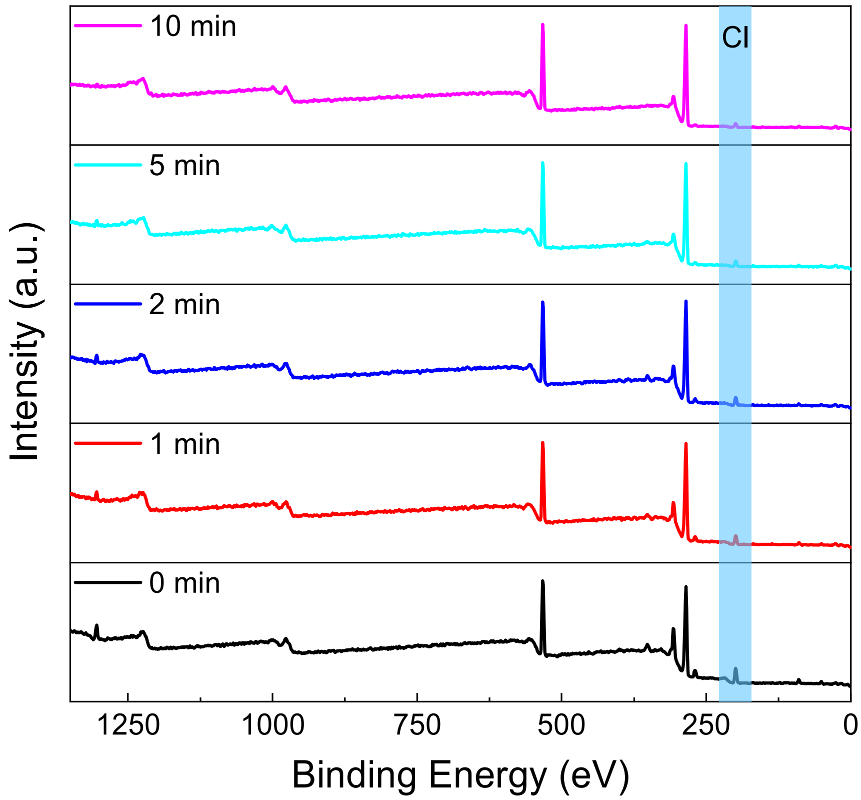

Supplementary Fig. 9. Full scan XPS spectrum of C-wood washed by water for different times. (The C-wood was grounded into powders. These powders were immersed into

water for different time. After that, the powders were obtained by filtration and dried at 105°C. As-obtained sample was divided into two ports. One port is for lifetime measurement and another port was used for determining the Cl content.)

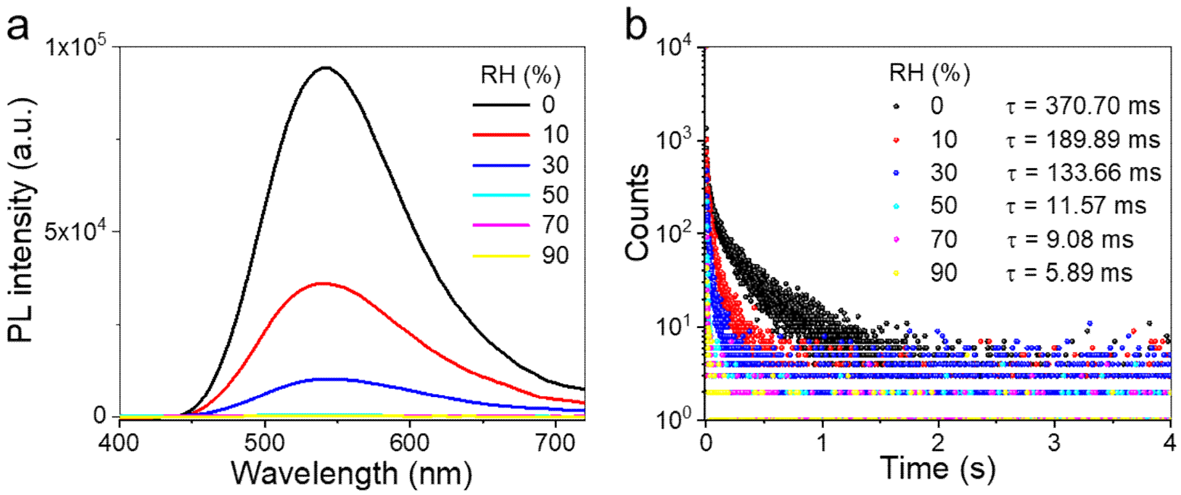

Supplementary Fig. 10. Phosphorescence properties of C-wood under conditions of different relative humidity. (a) Afterglow emission spectra of C-wood determined under different relative humidity conditions. (b) Lifetime decay profiles of C-wood determined under different relative humidity conditions. Excitation wavelength = 365 nm.

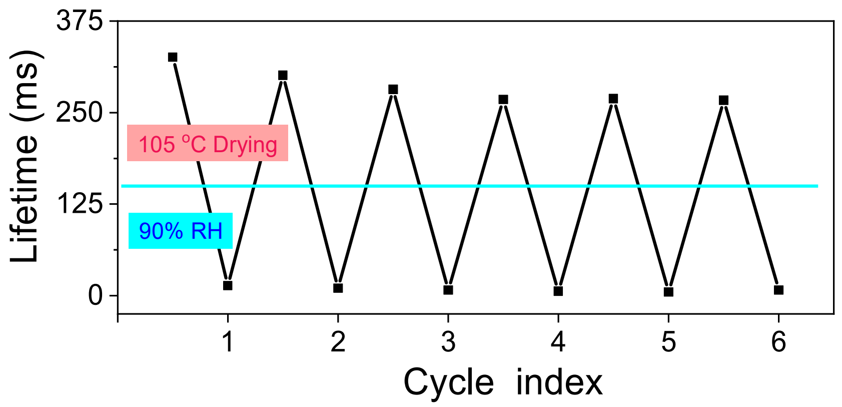

Supplementary Fig. 11. RTP lifetime of C-wood after multiple "humidifying-dehumidifying"

cycles; excitation wavelength = 365 nm.

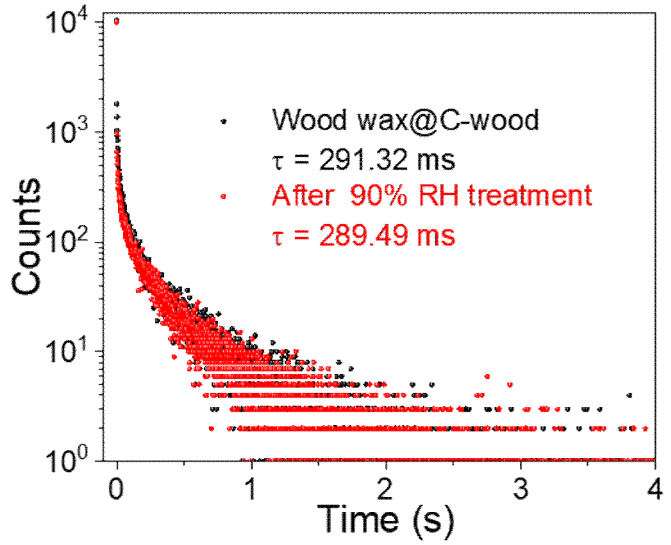

Supplementary Fig. 12. RTP decay profiles of wood wax@C-wood and wood wax@C-wood treated with 90% relative humidity (RH); excitation wavelength = 365 nm.

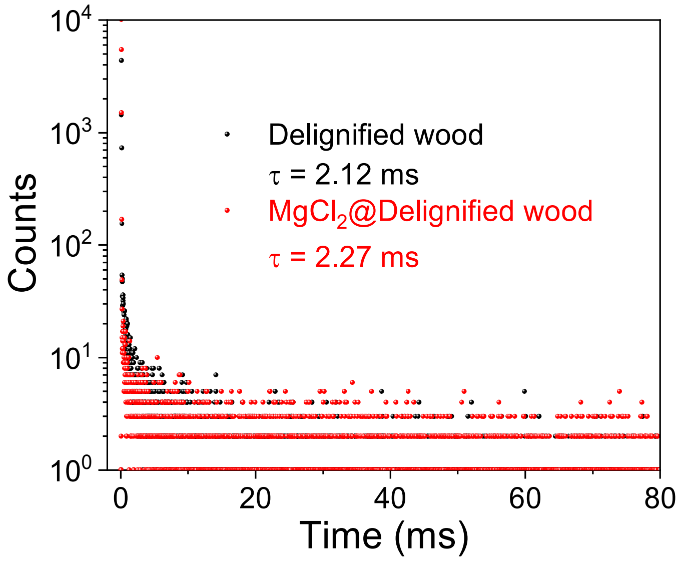

Supplementary Fig. 13. RTP decay profiles of delignified wood and $MgCl_2$@delignified

wood, excitation wavelength = 365 nm. (**Preparation of delignified wood**: The lignin removal solution was prepared by dissolving NaOH (2.5 mol L^{-1}) and Na_2SO_3 (0.4 mol L^{-1}) in deionized water. The wood slices (50 × 50 mm^2; thickness of 0.5 mm) were immersed in the lignin removal solution, boiled for 8 h, and then rinsed three times with hot deionized water to remove most of the chemicals. The pretreated wood slices were then placed on grids, which were suspended ~2 cm above a boiling aqueous solution of H_2O_2 (30 wt %). When the yellow color of the wood slices completely disappeared (~12 h), the samples were removed and rinsed with cold water to provide delignified wood (~0.21% residual lignin, determined by Klason method).

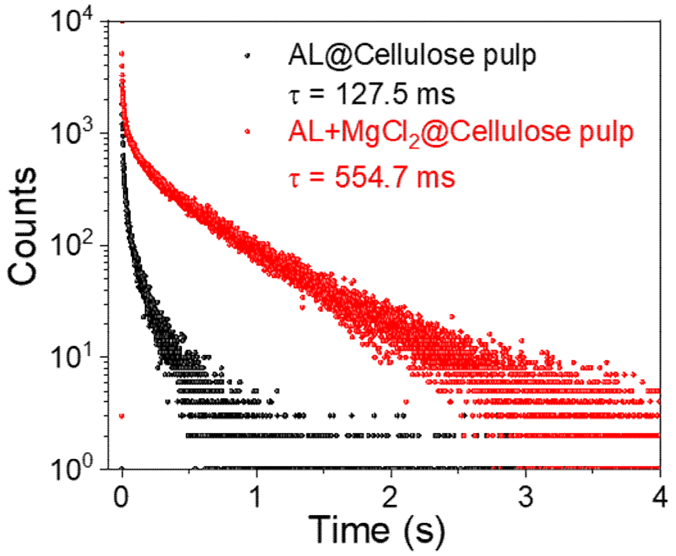

Supplementary Fig. 14. RTP decay profiles of AL@Cellulose pulp and AL + $MgCl_2$@Cellulose pulp, excitation wavelength = 365 nm. (**Preparation of AL @cellulose pulp**: The cellulose pulp was first treated with 5 mg mL^{-1} AL, and then it was dried at 105 ^oC to get AL @cellulose pulp; **Preparation of AL + $MgCl_2$@cellulose pulp**: AL @cellulose

pulp was treated with 1 mol L^{-1} $MgCl_2$ and dried at 105 °C to obtain AL + $MgCl_2$@cellulose pulp.)

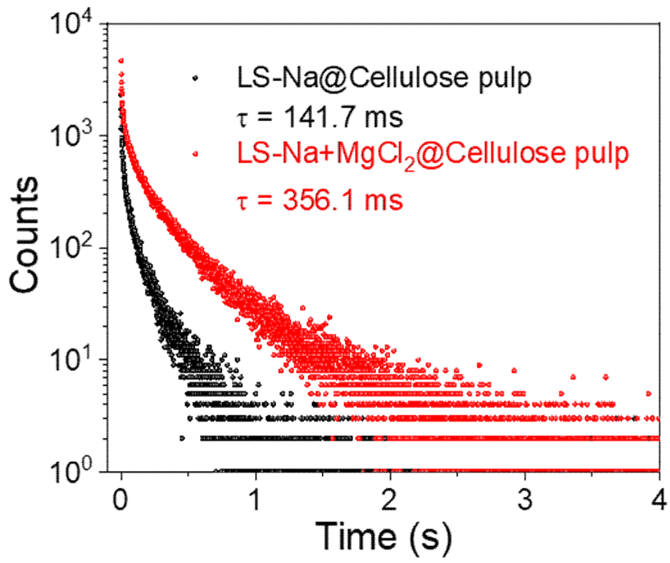

Supplementary Fig. 15. RTP decay profiles of LS-Na@Cellulose pulp and LS-Na + $MgCl_2$@Cellulose pulp, excitation wavelength = 365 nm. (**Preparation of LS-Na@cellulose pulp**: The cellulose pulp was first treated with 5 mg mL^{-1} LS-Na, and then it was dried at 105 °C to get LS-Na@cellulose pulp; **Preparation of LS-Na + $MgCl_2$@cellulose pulp**: LS-Na@cellulose pulp was treated with 1 mol L^{-1} $MgCl_2$ and dried at 105 °C to obtain LS-Na + $MgCl_2$@cellulose pulp.)

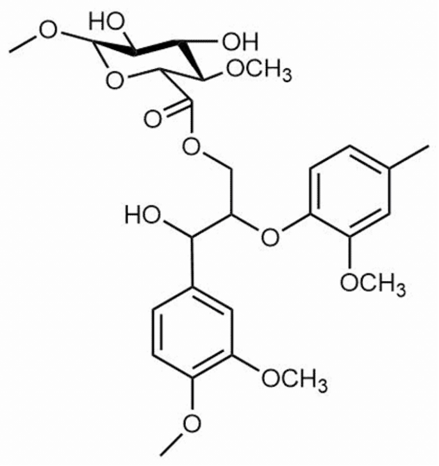

Supplementary Fig. 16. Structure of model molecule used for calculations involving the wood sources considered in this study.

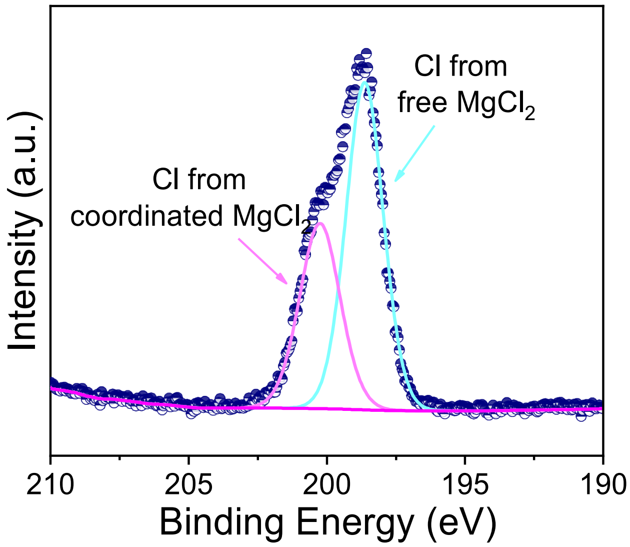

Supplementary Fig. 17. High resolution scans of Cl of C-wood.

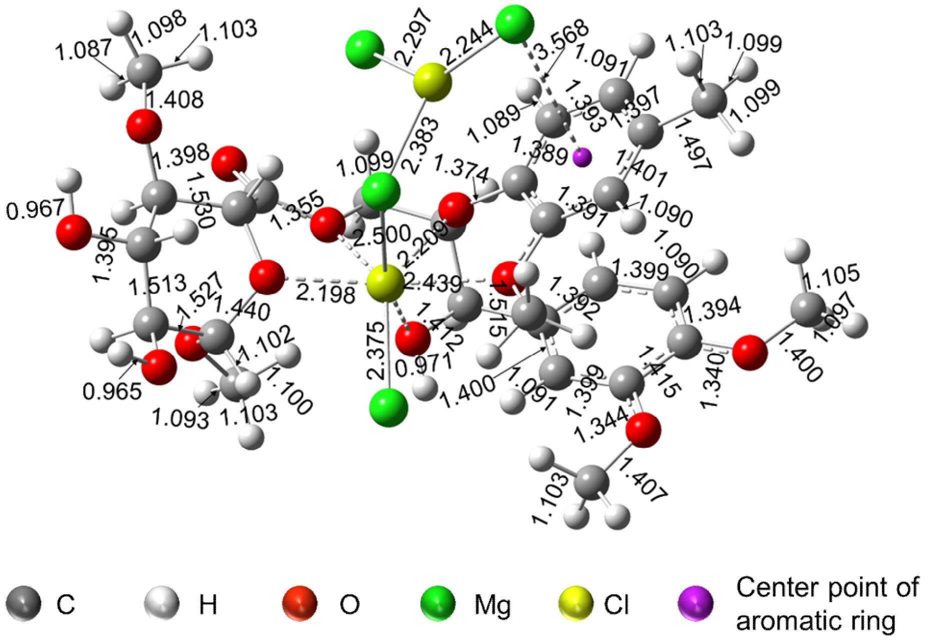

Supplementary Fig. 18. Structure of C-wood as inferred from theoretical calculations.

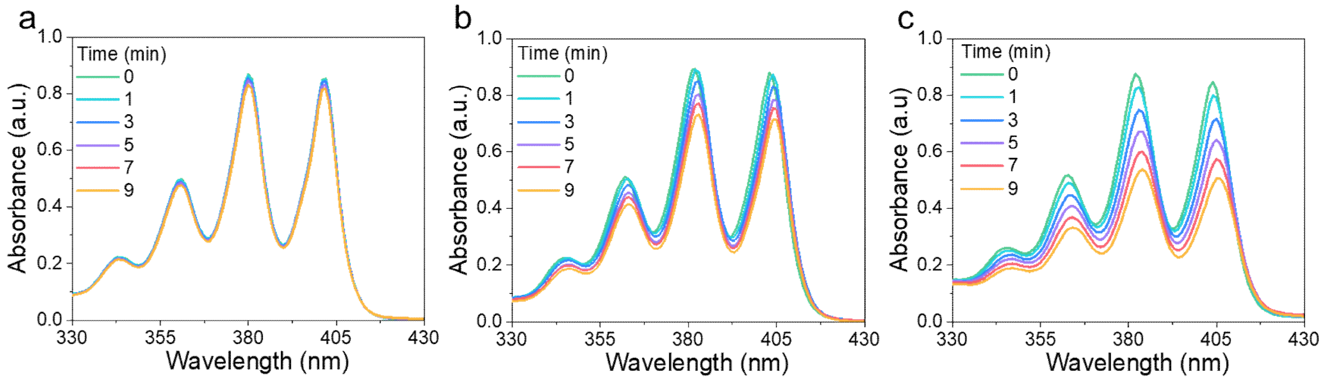

Supplementary Fig. 19. Time-dependent bleaching of ABDA caused by the $^1O_2$ generation of (a) blank control, (b) wood and (c) C-wood under 365 nm irradiation. (Concentration: 40 ppm (ABDA), UV light irradiation (5W, the distance from sample is 45cm)).

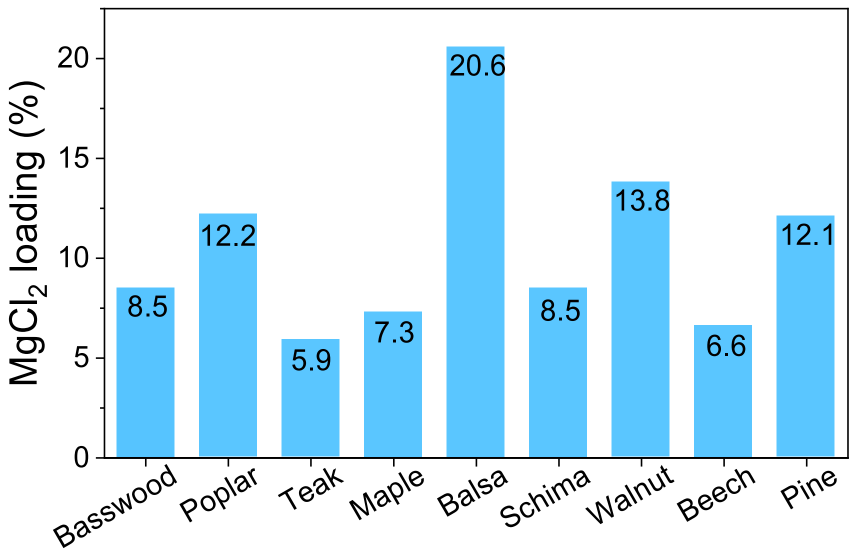

Supplementary Fig. 20. The loading of $MgCl_2$ (1M) in different wood.

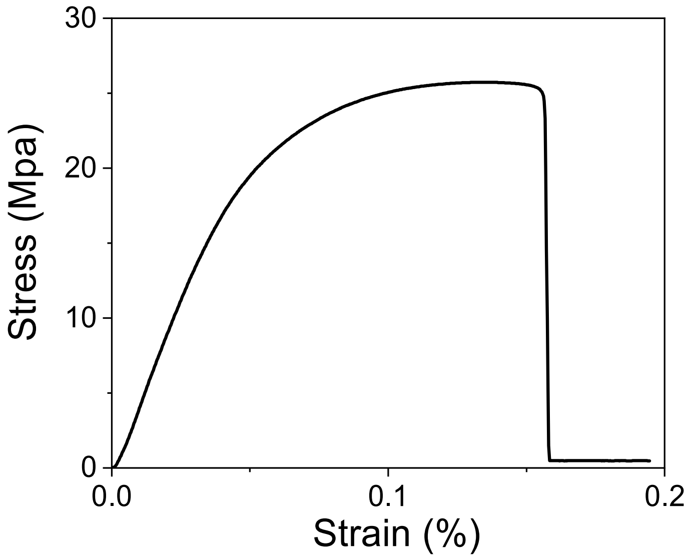

Supplementary Fig. 21. Tensile strength of printable fibers.

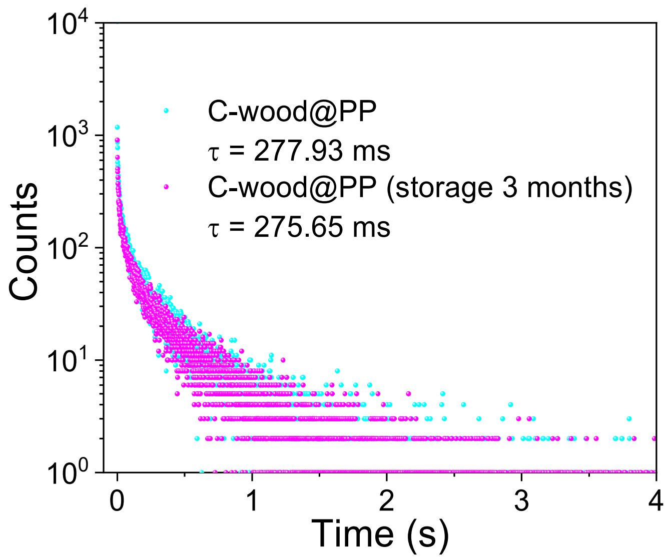

Supplementary Fig. 22. RTP decay profiles of C-wood@PP and C-wood@PP after storage 3 months, excitation wavelength = 365 nm.

Supplementary References

1. Grimme S, Bannwarth C, Shushkov P. A Robust and Accurate Tight-Binding Quantum Chemical Method for Structures, Vibrational Frequencies, and Noncovalent Interactions of Large Molecular Systems Parametrized for All spd-Block Elements (Z=1-86). *J. Chem. Theory Comput.* **13**, 1989-2009 (2017).
2. Bannwarth C, Ehlert S, Grimme S. GFN2-xTB-An Accurate and Broadly Parametrized Self-Consistent Tight-Binding Quantum Chemical Method with Multipole Electrostatics and Density-Dependent Dispersion Contributions. *J. Chem. Theory Comput.* **15**, 1652-1671 (2019).
3. Bannwarth C*, et al.* Extendedtight-bindingquantum chemistry methods. *Wiley Interdiscip. Rev. Comput. Mol. Sci.* **11**, e1493 (2021).
4. Adamo C, Barone V. Toward reliable density functional methods without adjustable parameters: The PBE0 model. *J. Chem. Phys.* **110**, 6158-6170 (1999).
5. Weigend F, Ahlrichs R. Balanced basis sets of split valence, triple zeta valence and quadruple zeta valence quality for H to Rn: Design and assessment of accuracy. *Phys. Chem. Chem. Phys.* **7**, 3297-3305 (2005).
6. Neese F. The ORCA program system. *Wiley Interdiscip. Rev. Comput. Mol. Sci.* **2**, 73-78 (2012).
7. Neese F. Software update: the ORCA program system, version 4.0. *Wiley Interdiscip. Rev. Comput. Mol. Sci.* **8**, e1327 (2018).
8. Humphrey W, Dalke A, Schulten K. VMD: Visual molecular dynamics. *J Mol. Model.* **14**, 33-38 (1996).
