# Supplementary material for: Room temperature phosphorescence from natural wood activated by external chloride anion treatment
Source: Nat Commun. 2023 May 5;14:2614. doi: 10.1038/s41467-023-37762-9 (PMC10162966; doi:10.1038/s41467-023-37762-9)
Supplement: Supplementary file 2 — Description of Additional Supplementary Files [file 41467_2023_37762_MOESM2_ESM.pdf]

Description of Additional Supplementary Files

File Name: Supplementary Movie 1
Description: Automatic manufacturing line of C-wood. (Notably, as-obtained C-wood can only give RTP emission after drying.)

File Name: Supplementary Movie 2
Description: The afterglow video of C-wood.
